# Nanoscale electrical property studies of individual GeSi quantum rings by conductive scanning probe microscopy

**DOI:** 10.1186/1556-276X-7-659

**Published:** 2012-11-29

**Authors:** Yi Lv, Jian Cui, Zuimin M Jiang, Xinju Yang

**Affiliations:** 1 State Key Laboratory of Surface Physics, Fudan University, Shanghai 200433, China

**Keywords:** GeSi quantum rings (QRs), electrical properties, SKM, CAFM, SCM

## Abstract

The nanoscale electrical properties of individual self-assembled GeSi quantum rings (QRs) were studied by scanning probe microscopy-based techniques. The surface potential distributions of individual GeSi QRs are obtained by scanning Kelvin microscopy (SKM). Ring-shaped work function distributions are observed, presenting that the QRs' rim has a larger work function than the QRs' central hole. By combining the SKM results with those obtained by conductive atomic force microscopy and scanning capacitance microscopy, the correlations between the surface potential, conductance, and carrier density distributions are revealed, and a possible interpretation for the QRs' conductance distributions is suggested.

## Background

Self-assembled semiconductor quantum rings (QRs) are an alternative type of quantum structures, which have received great interests in recent years for their unique properties and potential applications in nano-electronic devices [1-4]. It has been reported that the QRs have especially interesting characteristics due to the ring-shaped geometry, such as persistent current and A-B effects [5]. However, compared to the intensive theoretical studies on the ideal or lithographed QRs, the studies addressing the self-assembled QRs are relatively lacked. Furthermore, among the existing studies on self-assembled QRs, most of them are dealing with the growth mechanisms or electronic properties of QRs [6-11], while some of the studies are performed on the QRs' composition and strain distributions or atomic structures [12-16]. The electrical characteristics of the QRs, which are of vital importance to nano-electronic applications, have much less been concerned.

In recent decades, scanning probe microscopy (SPM)-based techniques have become effective means to investigate the electrical properties of individual quantum structures, which can provide non-averaged quantum properties [17,18]. For example, conductive atomic force microscopy (CAFM) enables us to investigate the conductive properties of individual quantum structures [19-21], while scanning Kelvin microscopy (SKM) [22] and scanning capacitance microscopy (SCM) [23] are valuable tools for measuring the surface potential and carrier density distributions of individual quantum structures. These techniques have already been performed to study the electrical properties of individual quantum dots [24-31], but the electrical property studies on individual QRs are still lacking. Up to now, there are only a few papers reported about the QRs' conductance distributions [32,33]. To gain further insight into the QRs' electrical properties as well as the intrinsic mechanisms, herein SKM is employed to investigate the surface potential distributions of individual GeSi QRs. Ring-shaped surface potential distributions of GeSi QRs are obtained, for the first time to our knowledge. By combining with the results obtained by CAFM and SCM, the correlations between the surface potential, conductance, and carrier density distributions are revealed, and a possible explanation for the QRs' conductance distribution is suggested.

## Methods

The GeSi QRs studied here were grown on a p-type Si(001) wafer (1 ~ 10 Ω cm) in a solid source molecular beam epitaxy (Riber EVA-32, France) system [6]. First, a 64-nm-thick Si buffer layer was deposited on the wafer at 640°C, followed by a 2.5-nm-thick Ge layer depositing at the same temperature. Then, a 3.2-nm-thick Si cap layer was deposited at the same temperature of 640°C to form GeSi QRs. The growth details as well as the formation mechanism of the QRs were discussed in our previous paper [6].

The electrical property measurements were performed on a Multimode V (Bruker Nano Surfaces, MA, USA) SPM instrument. The conductive properties of single QRs are measured by CAFM in contact mode under a DC bias. Pt-coated Si tips are applied in CAFM measurement, and a bias of −1 V is applied to the sample, while the tip is grounded. For SKM measurements, a voltage consisting of a DC bias with a small AC modulation is applied to the tip. During the scan, the DC bias is adjusted to be always equal to the contact potential difference (CPD) between the tip and the sample surface at each point through the feedback system, which forms the CPD image. SCM measures the capacitance variation with a small voltage variation, i.e., dC/dV. For a metal tip/oxide/semiconductor system, the dC/dV amplitude is inversely proportional to the carrier density in semiconductor. Thus, the carrier density distribution can be obtained from the dC/dV amplitude image. Their detailed operation principles can be found in previous reviews [17,18,22]. W_2_C-coated Si tips are employed in SKM and SCM measurements. Before CAFM and SKM measurements, the samples would be etched in diluted HF solution for 30 s to remove the native oxide layer which could keep the sample surface free-oxidized in the next 2-h when the experiments were performed in a flowing nitrogen atmosphere [34], and the measurements were mostly carried out during this time period. To exclude the influence of composition distribution, the sample was etched in a BPA solution (HF/H_2_O_2_/CH_3_COOH = 1:2:3) to remove the GeSi alloys with Ge ratio more than 6% [35,36], leaving a Si-dominated surface.

## Results and discussions

**Figure 1:**
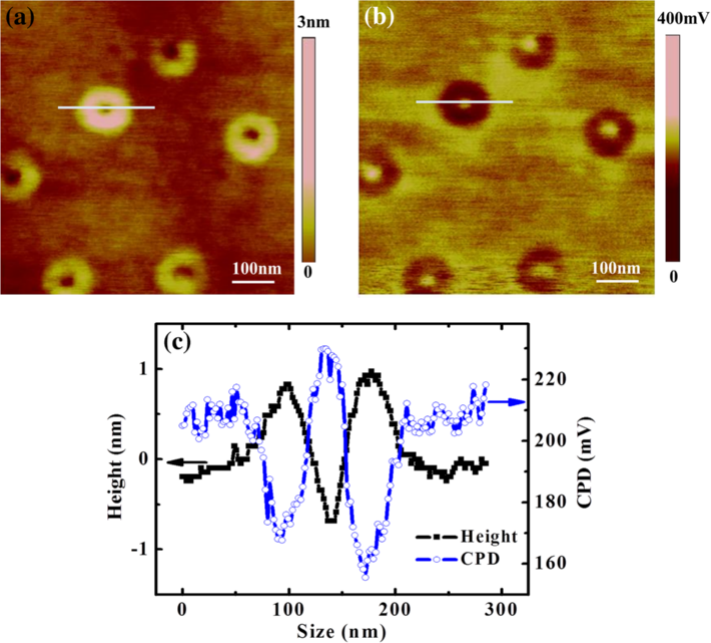
**SKM images of GeSi QRs. **The topography and potential images of individual GeSi QRs are shown in (**a**) and (**b**), respectively. Their height and potential profiles of an individual QR along the marked line are shown in (**c**).

The topography and CPD images of GeSi QRs are shown in Figure 1a,b, respectively. The height and CPD profiles of an individual QR along the marked line are shown in Figure 1c. It can be seen that the surface potential of the GeSi QRs exhibits a ring-shaped feature, showing that the QRs' rim has smaller CPD values than the QRs' central hole. In SKM, the measured CPD is defined as *Φ*_tip_ − *Φ*_sample_[37-39], where *Φ*_tip_ and *Φ*_sample_ are work functions of the tip and the sample, respectively. The conductive tip used in SKM was W_2_C-coated Si tip. Its work function is calibrated with HOPG sample whose work function is about 4.6 eV [37]. By analyzing the surface potential images of HOPG, the work function of W_2_C-coated tip used in this experiment is obtained to be about 4.54 eV. Also, we checked the work function of the PtIr-coated Si tip that was used in CAFM measurements. Its work function is about 4.85 eV which is in good agreement with the reported results [38]. Thus, the work functions of the QRs' rim and central hole, as well as the wetting layer, are obtained which are about 4.38, 4.31, and 4.34 eV, respectively. The results suggest that the QRs' central hole has a smaller work function than the QRs' rim, even smaller than the wetting layer.

**Figure 2:**
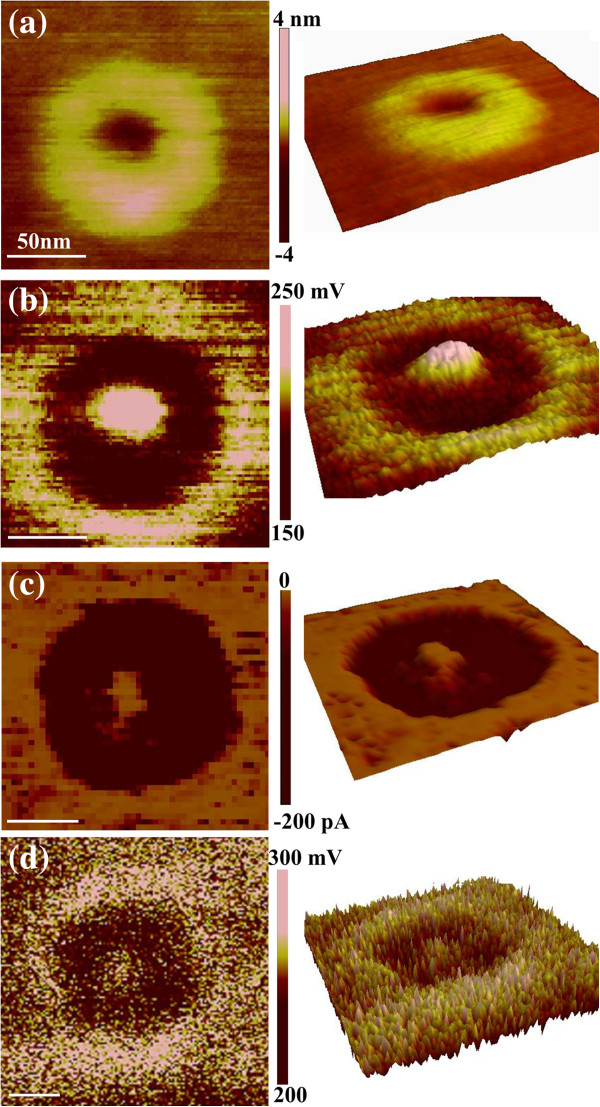
**SKM**, **CAFM, and SCM images **(**left column**) **and 3D views **(**right column**) **of an original GeSi QR. **(**a**) Height image measured simultaneously with the CPD image. (**b**) CPD image obtained at an AC modulation of 2 V and a lift height of 10 nm. (**c**) Current image measured at a sample bias of −1 V. (**d**) dC/dV amplitude image obtained by applying 2 V AC modulation to the sample. Each scale bar is 50 nm.

The current and carrier density properties of the above GeSi QRs are also measured by CAFM and SCM. The height, CPD, current, and dC/dV amplitude images of a typical QR are shown in Figure 2a,b,c,d, respectively. It should be mentioned that it is not the same QR measured in SKM, CAFM, and SCM. The geometrical shapes of the QRs are a little varied, but it does not influence the investigation of their distributions. Figure 2a shows that the QR has a typical ring structure in topography. Figure 2b presents a clear ring-shaped CPD distribution, just as that shown in Figure 1b. From the current image shown in Figure 2c, a ring-shaped current distribution can be clearly observed, demonstrating that the QRs' rim has a higher conductivity than the central hole. However, our previous studies on the composition distributions of GeSi QRs disclosed that the QRs' central hole has a much higher Ge content than the rim [33]. It indicates that the conductance distribution of GeSi QRs does not agree with their composition distribution, which is however not well understood yet. From the SCM results (Figure 2d), it can be seen that the dC/dV amplitude obtained at the QRs' rim is a little smaller than both obtained at the central hole and the wetting layer, also exhibiting a ring-shaped distribution. Since smaller dC/dV amplitude corresponds to higher carrier density in the semiconductor for the tip/oxide/semiconductor structure [17,18], our results suggest that the carrier density in the QRs' rim is higher than that in the central hole. The averaged values of the dC/dV amplitude are about 220, 260, and 250 mV for the QRs' rim, central hole, and wetting layer, respectively.

**Figure 3:**
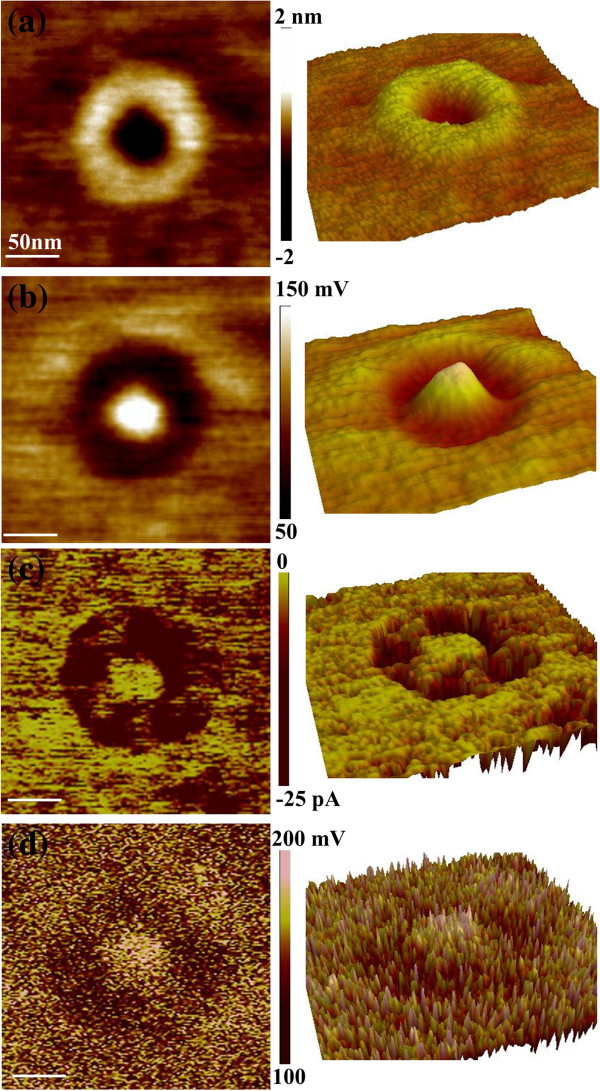
**SKM, CAFM, and SCM images **(**left column**) **and 3D views **(**right column**) **of BPA**-**etched GeSi QR. **(**a**) Height image measured simultaneously with the CPD image, (**b**) CPD image obtained at an AC modulation of 2 V and a lift height of 10 nm. (**c**) Current image measured at a sample bias of −1 V. (**d**) dC/dV amplitude image obtained by applying 2 V AC modulation to the sample. Each scale bar is 50 nm.

From the above results, all the QRs' surface potential, conductance, and carrier density exhibit ring-shaped distributions, just like their geometrical shapes. The QRs' rim has a larger work function, a higher conductivity, and a larger carrier density than both the central hole and the wetting layer. Thus, one may suppose that the QRs' geometrical shape has an important impact on their electrical properties. To further check this viewpoint, the electrical properties of the BPA-etched QRs are measured. Dipping the GeSi QRs in BPA solution can remove the GeSi alloys with Ge content larger than 6% [35,36], leaving a surface dominated by Si atoms. Thus, for the BPA-etched QRs, the influences of composition and strain distributions become much weaker, while the influence of geometrical shape on the electrical properties still needs to be concerned. The height, CPD, current, and dC/dV amplitude images of a typical QR after BPA etching are shown in Figures 3a,b,c,d, respectively. The geometrical structures of the BPA-etched QRs still exhibited ring-shaped features, with a much more broadened and deeper central hole. On the other hand, the surface potential of the BPA-etched QRs also exhibits a similar ring-shaped distribution to that of the original QRs, which has lower surface potentials at the rim than at the central hole and the wetting layer. By a statistical analysis over a number of QRs, the work functions of the rim, the central hole, and the wetting layer for BPA-etched QRs are obtained to be about 4.48, 4.37, and 4.43 eV, respectively. The CPD difference (0.11 eV) between the rim and the central hole for the BPA-etched QRs is larger than that for the original QRs (0.07 eV), which may be due to the QRs' geometrical or compositional change after BPA etching. Nevertheless, the ring-shaped CPD distribution is unchanged after BPA etching. Also, the BPA-etched QRs' current distribution presents a ring-shaped feature, similar to the original one (Figure 2c). Only the current values of the BPA-etched QRs are much smaller than those of the original QRs; this is because most of the conductive GeSi alloys are etched away. From the SCM results, a similar ring-shaped dC/dV amplitude distribution can be observed. The QRs' rim has lower dC/dV amplitude than the QRs' central hole, indicating that the BPA-etched QRs' rim still has a higher carrier density than the QRs' central hole. The averaged values of the dC/dV amplitude are 130, 170, and 150 mV for the QRs' rim, central hole, and wetting layer, respectively. The difference of the dC/dV amplitude between the rim and the central hole is also larger for BPA-etched QRs than that for original QRs, as the carrier density is inversely proportional to the dC/dV amplitude. All the above results indicate that the composition distribution has little impact on their electrical property distributions (i.e., ring-shaped distributions), and it only changes the absolute values of the electrical properties. In addition, as the BPA etching can remove most of the GeSi alloys with Ge content larger than 6%, the strain effect becomes considerably weak for BPA-etched QRs, which still present ring-shaped distributions. Therefore, the change of composition and strain distributions does not modify the ring-shaped electrical property distribution. Thus, it can be identified that the composition and strain distributions could not be the main factors to determine the QRs' electrical properties. Furthermore, from the current, surface potential, and dC/dV amplitude images, it can be observed that the QRs with different sizes have similar ring-shaped distributions, except the absolute values are varied. Hence, the ring-shaped electrical property distributions also could not be attributed to the size effects. On the other hand, as all the QRs which exhibit ring-shaped electrical property distributions have ring-shaped geometry, it seems to be reasonable to suppose that the topographic shape should have vital influence on QRs' electrical property distributions, while size, composition, and strain only influence the values.

**Figure 4:**
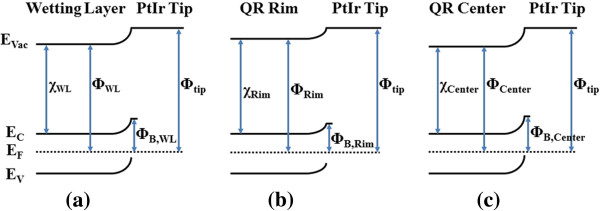
**Schematic energy band diagrams of the interface. **Between the AFM tip and the wetting layer (**a**), between the AFM tip and the QR's rim (**b**), and between the AFM tip and the QRs' central hole (**c**). The Schottky barrier height at the interface is termed as *Φ*_B,WL _for the wetting layer, *Φ*_B,Rim _for the QR's rim, and *Φ*_B,Center _for the QRs' central hole, respectively.

The reason why the QRs' topographic shape has an important impact on their electrical properties is not clear yet. For GeSi QDs, it is known that the QD structure can confine holes due to its composition variation. But for QRs, the holes should be confined to the whole QR structure, especially to the QRs' center due to it owns high-Ge content compared to the ring and wetting layer, if only considering the composition variation. But in our experiments, no current and lowest carrier density was measured at the QRs' center. Thus, the topographic factor should influence the carrier density distribution, though its origin is not understood yet. In previous theoretical studies dealing with ideal QRs [10] or InAs/GaAs QRs [5,40,41], it was found that either ring-shaped adiabatic potential [5] or carrier probability density [10,40,41] was achieved by considering the QRs' geometrical parameters, as well as strain [5], composition gradient [40], or piezoelectric potential [5,41], etc. Though, for a realistic QR, the distribution of carrier density would be much complex as many factors should be taken into consideration, the main feature of the ring-shaped potential and carrier density could be expected. Since no reference has been found to report the electronic properties of GeSi QRs and the above considerations on InAs QRs are consistent with our SKM and SCM results, we suggest the QRs' ring-shaped surface potential and carrier density distributions are attributed to their geometrical shapes follows the above concept. The correlation between the conductance distribution and the carrier density distribution is direct, i.e., higher carrier density resulting in larger conductivity; our SCM results did agree with the QRs' conductance distribution. The correlation between the conductance distribution and the surface potential distribution could be described with the viewpoint of electron barrier height at the interface between the tip and the measured surface, which was introduced by Lochthofen et al. to interpret the higher conductivity of the V-defects in GaN film [42]. The schematic band diagrams of the interfaces between the PtIr tip and the wetting layer, the rim of the QR, and the central hole of the QR are shown in Figure 4a,b,c, respectively. Since in our CAFM experiments the sample is −1 V biased with respect to the grounded tip, the electrons flow from the sample to the tip. From the results of SKM, the electron barrier height for the QRs' rim (*Φ*_B,Rim_) is found to be lower than that for the QRs' central hole (*Φ*_B,Center_) and the wetting layer (*Φ*_B,WL_). This is consistent with the CAFM results which show that the QRs' rim is more conductive than the QRs' central hole and wetting layer.

Based on the above considerations, a possible explanation of the QRs' electrical properties was suggested: the ring-shaped geometry determined that the QRs' rim has a lower barrier height with the tip and a higher carrier density, resulting in a higher conductivity at the rim, compared to the central hole and the wetting layer.

## Conclusion

In summary, the electrical properties of individual GeSi QRs were investigated by SKM, CAFM, and SCM. Ring-shaped surface potential, conductance, and carrier density distributions are achieved on individual original and BPA-etched GeSi QRs. Based on these results, it can be suggested that the ring-shaped surface potential distribution and/or carrier density distribution are the important contributors to QRs' conductance distribution.

## Competing interests

The authors declare that they have no competing interests.

## Authors’ contributions

YL carried out the experiments. JC prepared the samples. YL and XJY interpreted the results and wrote the manuscript. ZMJ helped in the sample preparation and discussions. All authors read and approved the final manuscript.
